# Chemical Characterization and Biological Properties of Leguminous Honey

**DOI:** 10.3390/antiox13040482

**Published:** 2024-04-18

**Authors:** Florinda Fratianni, Giuseppe Amato, Maria Neve Ombra, Vincenzo De Feo, Filomena Nazzaro, Beatrice De Giulio

**Affiliations:** 1 Institute of Food Science, CNR-ISA, Via Roma 64, 83100 Avellino, Italy; fratianni@isa.cnr.it (F.F.); nives.ombra@isa.cnr.it (M.N.O.); defeo@unisa.it (V.D.F.); beatrice.degiulio@isa.cnr.it (B.D.G.); 2 Department of Pharmacy, University of Salerno, Via Giovanni Paolo II, 84084 Fisciano, Italy; gamato@unisa.it

**Keywords:** honey, polyphenols, VOCs, antioxidant activity, neurodegenerative diseases, anti-inflammatory activity

## Abstract

Honey can beneficially act against different human diseases, helping our body to improve its health. The aim of the present study was first to increase knowledge of some biochemical characteristics (amount and composition of polyphenols and volatile organic compounds, vitamin C content) of five Italian legume honeys (alfalfa, astragalus, carob, indigo, and sainfoin). Furthermore, we evaluated their potential health properties by studying their antioxidant and in vitro anti-inflammatory activities and in vitro inhibitory effects on three enzymes involved in neurodegenerative diseases (acetylcholinesterase, butyrylcholinesterase, and tyrosinase). Alfalfa honey showed the highest total polyphenol content (TPC) (408 μg g^−1^ of product). Indigo honey showed the lowest TPC (110 μg g^−1^ of product). The antioxidant activity was noteworthy, especially in the case of sainfoin honey (IC_50_ = 6.08 mg), which also exhibited excellent inhibitory action against butyrylcholinesterase (74%). Finally, the correlation between the biochemical and functional results allowed us to identify classes of molecules, or even single molecules, present in these five honeys, which are capable of influencing the properties indicated above.

## 1. Introduction

Honey, obtained from the work of bees, symbolizes the product par excellence. It has always been linked to the history of man, and so much so that its properties—but above all, its beneficial effects—have been used since almost the dawn of civilization. The presence of bioactive molecules, such as polyphenols (in particular, phenolic acids such as gallic, *p*-coumaric, caffeic, and ferulic, chlorogenic acids, and flavonoids such as catechin, quercetin, naringenin, kaempferol, etc.), vitamins (in particular, vitamin C), enzymes, sugars, and, to a lesser extent, volatile compounds, influences, in general, the health properties of honey [1,2]. Many important biological functions are attributed to honey, such as anti-inflammatory [3], antioxidant [4], antibacterial, prebiotic [5,6], and immune-stimulant properties [7]. Furthermore, honey may exhibit cardio protective and hypocholesterolemic effects [8,9].

In recent years, some studies have evaluated the beneficial effects attributable to honey and other bee products in combating some of the most critical neurodegenerative diseases (NDDs), such as Alzheimer’s disease (AD) and Parkinson’s disease (PD), which are a consequence of neuronal death occurring in different parts of the brain and the central nervous system, mainly manifesting in older people [10,11].

Such pathologies involve some enzymes, such as acetylcholinesterase (AChE) and butyrylcholinesterase (BChE), responsible for the degradation of neurotransmitters [12] and tyrosinase. Inhibition of AChE and BChE may be helpful in the treatment of Alzheimer’s disease [13]; similarly, Parkinson’s disease can also be fought by inhibiting the action of the tyrosinase enzyme [14]. Several NDDs can also be connected to neuroinflammation and the amassing of free radicals prior to neurodegeneration [15], and, through a decrease in antioxidants, to the final nerve death [16]. The neuroprotective effects of honey are believed to be exercised at various stages of the neurodegenerative process. Therefore, honey could be a potential candidate for the diminishing of oxidative stress and inflammation and, concurrently, the meliorating of spatial memory performance [17]. Such effects could result from the presence and synergistic action of bioactive compounds, including polyphenols (flavonoids and acidic phenols) [18,19,20,21,22]. Depending on the botanical origin of honey, some secondary metabolites, such as polyphenols, may also lead to different biological activities of honey [23]. The volatile profile is another significant characteristic of honey. Volatile organic compounds (VOCs), present in the honey at very low concentrations as a complex mixture, belong to different classes of chemical compounds. VOCs, contributing to the aroma of honey, vary in quality and quantity due to several factors, such as the nectar-providing plant species, the action of honeybees and microorganisms, the geographical area, the environment, processing, and storage conditions, as well as the harvesting technology. Each volatile compound in the mixture may donate a different aroma, taste, and function, leading to the uniqueness of honeys [24,25,26]. Nowadays, the analysis of honey VOCs is a focus of enormous interest in apiculture since it can help to discriminate and characterize a product. Even though many VOCs can be present in the honey samples, some are specific, giving certain kinds of honey a particular fingerprint.

Consequently, knowledge of the VOCs profile and the floral origins of a particular honey can help standardize its quality and avoid fraudulent labeling of the product. Although only present in low concentrations, VOCs could also contribute to the biological activities of honey, especially the antioxidant effect, due to their natural ability to scavenge radicals [27,28]. Monofloral and multi-floral honeys exhibit different flavors, chemical compositions, and therapeutic properties. Legume honey is produced by bees that primarily collect nectar from leguminous plants, such as clover, acacia, and alfalfa, representing the most diffused types of legume honey. However, legume species’ biodiversity is enormous, and their heritage can further increase the benefit even of neglected leguminous species. In Italy, around 100,000 hectares are planted with legumes, which yield about 190,000 tons of mainly chickpeas, lentils, and peas [https://blog.wetipico.it/i-legumi-italiani-alla-scoperta-della-loro-importanza/ (last accessed: 7 February 2024)]. Italian production differs in quality and cultural importance. The recommencement of legume cultivation, particularly in some areas of the Italian territory, such as the central and southern regions, increased the production of ancient varieties, which can contribute to the safeguarding of plant biodiversity and the discovery of other legume derivatives, such as honey, with intriguing health properties. Previous work has demonstrated that some kinds of legume honey are effective in contrasting the mature biofilms of some pathogens, which is of interest to food and health, and, concurrently, in stimulating the growth and some functional properties of different probiotic bacteria [29]. Therefore, the objectives of the present study were as follows: (i) to increase the knowledge of some biochemical characteristics (total polyphenols, the qualitative–quantitative profile of polyphenols, the dosage of vitamin C content, and VOCs profiles) of five legume honeys; (ii) to evaluate their potential healthy properties by studying their in vitro antioxidant, anti-inflammatory, and inhibitory activity with respect to AChE, BChE, and tyrosinase. Finally, by correlating the biochemical and functional results, this paper tried to understand the influence that classes of molecules, or even single molecules, identified in the honey through UPLC and HS SPME/GC-MS analyses, can exert on the indicated properties.

## 2. Materials and Methods

### 2.1. Sample Collection

Five commercial organic Italian legume honeys—alfalfa (*Medicago sativa* L., from Tuscany), astragalus (*Astragalus nebrodensis* (Guss.) Strobl., from Sicily), carob (*Ceratonia silique* L., from Sicily), indigo (*Indigofera tinctoria* L., from Lombardy), and sainfoin (*Onobrychis viciifolia* Scop., from Abruzzo)—were analyzed. The companies indicated the monofloral character of the honeys, respected Italian law 179 of 2004, which also legislates on the floral or vegetable origin of a product if the product is wholly or mainly obtained from the indicated plant, and possesses its organoleptic, physicochemical, and microscopic characteristics. Three packages for each honey have been analyzed; all samples belonged to the same year of production. No honey crystallization appeared at the time of purchase. Samples were kept dark at room temperature (20 °C) until the analyses. 

### 2.2. Reagents and Chemicals

The analyses were carried using ultra-pure water from a Milli-Q system (Millipore, Bedford, MA, USA) with a resistivity of 18 MΩ*cm at 25 °C. Chemicals, standards, and reagents were from Sigma-Aldrich (St. Louis, MO, USA). Folin–Ciocalteu’s reagent was purchased from Carlo Erba (Cornaredo, Milano, Italy). 

As a GC carrier gas, helium was used at a purity of 99.999% (Rivoira, Milan, Italy). The HS-SPME fibers (DVB/CAR/PDMS; 50/30 μm, 1 cm) and the glass vials were from Supelco (Bellofonte, PA, USA). The capillary GC-MS column HP-Innowax (30 m × 0.25 mm × 0.5 μm) was purchased from Agilent J&W (Agilent Technologies Inc., Santa Clara, CA, USA). 

### 2.3. Biochemical Analyses

Following the method of Fratianni et al. [29], an aliquot from each of the three samples of honey was vigorously mixed with Milli-Q water (1:4 *w*/*v*), filtered (0.45 µm; Millipore, Milano, Italy), and subjected to the biochemical analyses.

#### 2.3.1. Total Polyphenol Content

Total polyphenol content (TPC) was assessed at room temperature using the Folin–Ciocalteu phenol reagent [6]. A UV/Vis spectrophotometer measured the absorbance at 760 nm (Cary Varian, Palo Alto, CA, USA). Results were expressed as µg of gallic acid (used as standard) equivalents/g of honey ± standard deviation (SD). The concentration range for the standard curve (y = 0.0134x + 0.0015; R^2^ = 0.979) was produced between 34.02 and 340.2 µg.

#### 2.3.2. Polyphenols Profile

An ACQUITY Ultra Performance LC™ system (Waters, Milford, MA, USA) linked to a PDA 2996 photodiode array detector (Waters) was used for ultra-high-performance liquid chromatography (UPLC) analyses [30]. The instruments were controlled through the Empower software EM5B01629. Before analysis, 0.45 μm of the samples and the standards (previously dissolved in methanol) was filtered. Run conditions: mobile phase 7.5 mM acetic acid (solvent A) and acetonitrile (solvent B) at a flow rate of 250 μL min^−1^; gradient elution: 5% B for 0.8 min, then 5–20% B over 5.2 min, isocratic 20% B for 0.5 min, 20–30% B for 1 min, isocratic 30% B for 0.2 min, 30–50% B over 2.3 min, 50–100% B over 1 min, isocratic 100% B for 1 min, and finally 100–5% B over 0.5 min; column assessment under the initial conditions for 2.5 min; pressure from 6000 to 8000 psi; T = 30 °C; column: reversed-phase column (BEH C18 1.7 μm, 2.1 × 100 mm, Waters); LC detector (scanning range: 210–400 nm, resolution: 1.2 nm); injection volume: 5 μL.

#### 2.3.3. Ascorbic Acid Content

With some modifications, the ascorbic acid was determined following the reduction method of the dye 2,6-dichlorophenolindophenol (DCPIP) [31]. A 0.3 mg mL^−1^ DCPIP solution was added to each sample, containing ascorbic acid in a 2.5% metaphosphoric acid and citrate–acetate buffer, and the absorbance was measured at 520 nm after 45 s. The tests were performed in triplicate. The standard curve (y = 7.0614x + 0.0722; R^2^ = 0.9885) was used to calculate results, which were expressed as mg of ascorbic acid 100 g^−1^ of the sample ± SD.

### 2.4. Antioxidant Activity

#### 2.4.1. Diphenyl-1-Picrylhydrazyl (DPPH) Test

Free-radical-scavenging activity was determined in microplates through the diphenyl-1-picrylhydrazyl (DPPH) test (29). Samples (15.15 μL) were mixed with 303 μL of a methanol solution of DPPH (153 mM). The absorbance was measured at λ = 517 nm (Cary Varian). The absorbance of the DPPH radical, without the sample, constituted the basis. The amount of samples required to inhibit the activity of 1 mL of DPPH at 50% specified the IC_50_ (mg). The tests were performed in triplicate, and the results were expressed as the mean ± SD.

#### 2.4.2. 2,2′Azino-Bis (3-Ethylbenzothiazoline-6-sulfonic acid) (ABTS) Test

The antioxidant activity of the honey, a key factor in its potential health benefits, was assessed through the 2,2′Azino-bis (3-ethylbenzothiazoline-6-sulfonic acid) (ABTS) test [32]. The measure of 2.5 mM of Trolox^®^ (methanolic daily preparation) constituted the antioxidant stock standard. The ABTS and potassium persulfate were dissolved in distilled water (final concentration of 7 and 2.45 mM, respectively). The resulting mixed solution was kept in the dark at room temperature before its use to produce the ABTS radical (ABTS+). Thereafter, the ABTS radical solution was diluted with deionized water to reach a final absorbance (= 1.00) at λ 734 nm. Lastly, samples (final concentrations: 0.0001–0.0100 mg. mL^−1^) or Trolox^®^ standard (final concentration: 0–20 mM) were added to the diluted ABTS+ solution; the absorbance was read 6 min after mixing. The average of triplicate determinations was expressed as the samples μmoL Trolox^®^ equivalent antioxidant capacity (TEAC) ± SD.

### 2.5. Inhibitory Effect of the Honey against Bovine Serum Albumin Degradation

The assay of the inhibition of serum bovine albumin denaturation, a precise method with which to evaluate in vitro anti-inflammatory activity, was used following the method of Fratianni et al. [33]. A stock solution of bovine serum albumin (BSA, 96% purity) was dissolved in 0.05 M Tris–phosphate buffer saline solution to obtain a final concentration of 0.5% (*w*/*v*); the pH was adjusted to 6.5 using glacial acetic acid. The reaction mixture (5 mL) contained 2.8 mL of phosphate Tris–phosphate buffer saline solution, 0.2 mL of BSA, and 2 mL of varying amounts (5, 10, 20, 30 μg) of honey. One ml of BSA containing methanol represented the control. After 5 min of heating at 72 °C, samples were cooled. The absorbance was calculated at 660 nm by a UV/Vis spectrophotometer (Cary Varian). The extract concentration for the 50% inhibition (IC_50_) of the BSA denaturation was determined compared with the control, using diclofenac sodium (1 mg mL^−1^) as the positive control.

### 2.6. Cholinesterase Inhibitory Activities

Inhibitory activities with respect to acetylcholinesterase (AChE) and butyrylcholinesterase (BChE) were determined via the spectrophotometric method of Ellman et al. [34]. Acetylcholine (ACh) was used as the substrate to evaluate the inhibition of AChE. A reaction mixture containing 550 μL of sodium phosphate buffer 0.1 M (pH 8.0), 50 μL of the honey sample, and different amounts (from 5 to 20 ng) of AChE (from *Electrophorus electricus* 1000 units mg ^−1^) was used. The reaction mixture was incubated at room temperature for 15 min. All samples and the positive control (galantamine) were dissolved in 10% dimethyl sulfoxide (DMSO). The formation, at 412 nm for 10 min of the yellow 5-thio-2-nitrobenzoate anion, resulting from the reaction of 5, 5-dithio-bis-2-nitrobenzoic acid (DTNB, 10 µL) with Ach (10 μL), released by the hydrolysis of Ach, indicated the enzyme activity. The inhibitory activity against BChE was evaluated through the use of butyrylthiocholine (BCh) as a substrate. The reaction mixture contained the following: 550 μL K-phosphate buffer 0.1 M (pH 7.0), 50 μL of the sample, and an amount of equine serum BchE (≥10 units/mg protein) ranging from 10 to 50 ng. The mixture was incubated at room temperature. After 15 min, the substrate BCh and the DTNB were added. The hydrolysis of BCh was monitored at 412 nm through the formation of the yellow 5-thio-2-nitrobenzoate anion for 10 min, resulting from the reaction of DTNB (10 μL) with butyrylcholine (10 μL), released by the enzymatic hydrolysis of BCh. The reactions were performed in triplicate in 96-well microtiter plates. The following equation allowed us to calculate the percent inhibition:

% inhibition = 1 − (B − b) × 100/(A − a) %inhibition = 1 − (B − b) × 100/(A − a),

where B is an initial enzyme reaction with the sample, and b is an initial reaction with the sample but without the enzyme; A is an initial reaction with the enzyme, and a represents an initial reaction without the enzyme. Determinations were performed in triplicate and results were expressed as the mean  ± SD. AChE- and BChE-inhibitory activities were also expressed in terms of the IC_50_ value (mg required for inhibition at 50% the hydrolysis of the substrate).

### 2.7. Tyrosinase Inhibition Assay

Honey samples were diluted (1:1) in dimethyl sulfoxide (DMSO) [35]. The reaction mixture was prepared by filling the 96-microtiter plate with the phosphate buffer (70 μL, pH 6.8), 10 units mL^−1^, and the sample. After a five-min incubation at 37 °C, L-tyrosine (or 0.5 mM l-dioxyphenylalanine (l-DOPA)) was included. The plate was read instantaneously at λ = 492 nm and at λ = 475 nm after 10 min of incubation at 37 °C. Kojic acid and phosphate buffer represented the positive control and the blank, respectively. The percentage of inhibition was calculated following the formula:

% I = [(Ab − As)/Ab × 100] %I = [(Ab − As)/Ab × 100],

where Ab is the absorbance of the blank sample at T10′, and As is the sample absorption at T10–sample absorbance at T0 (T0: beginning; T10: 10 min). Determinations were performed in triplicate and results are expressed as the mean  ±  SD. The inhibitory activity with respect to tyrosinase was also evaluated in terms of IC_50_—that is, the concentration of samples giving a 50% inhibition of tyrosinase activity. This evaluation was performed via interpolation of the concentration–response curves. The tests were executed in triplicate for each sample and for the control.

### 2.8. Volatile Organic Compounds Analysis

#### 2.8.1. Sample Preparation and HS SPME Procedure

The VOCs profile was obtained using the HS-SPME coupled to the gas chromatography-–mass spectrometry (GC-MS) method described by Baroni et al., with some modifications [36]. For the sample preparation, 3 g of honey was mixed with 1 g of NaCl and 3 mL of Milli-Q water into a 20 mL screw-on-cap HS amber vial. To assure the analytical reproducibility, in each sample vial, 5 µL from a stock solution of 1-octanol (initial concentration of 50 μg mL^−1^), used as internal standard (IS), was added. Vials were then hermetically sealed with a Teflon septum and aluminum cap (Chromacol, Hertfordshire, UK) and mixed using a vortex for a few min prior to analysis. The equilibration time and temperature were 15 min and 60 °C, respectively. The extraction and injection steps were automatically performed using an autosampler MPS 2 (Gerstel, Mülheim, Germany). The SPME fiber (DVB/CAR/PDMS; 50/30 μm, 1 cm) was then automatically introduced into the HS through the septum and exposed to the vial HS for 30 min to allow for the adsorption of VOCs onto the fiber surface. The sample was continually stirred to improve the extraction efficiency. The HS-SPME fibers, prior to their first use, were conditioned as suggested by the manufacturer but below the maximum recommended temperature. The fibers were conditioned before the initial daily analysis for 5 min at the operating temperature of the GC injector port, and the blank level was verified.

#### 2.8.2. Gas Chromatography–Quadrupole Mass Spectrometry Analysis (GC–qMS) 

VOCs analysis was carried out by inserting the HS-SPME fiber into the injector port of a gas chromatographer Agilent, model GC 7890A, coupled with a mass spectrometer 5975C (system from Agilent Technologies, Santa Clara, CA, USA). VOCs were thermally desorbed for 5 min at 250 °C and directly transferred to a capillary column HP-Innowax for the analysis. The GC oven temperature program was initially set at 60 °C for 5 min, then increased to 200 °C at 6 °C min^−1^, and finally maintained until 240 °C at 15 °C min^−1^ and held at this temperature for 5 min. Mass spectra were acquired at an ionization energy of 70 eV, and VOCs were detected via mass selective detector. The detector operated in a mass range between 30 and 300 u with a scanning speed of 2.7 scans/s. The identification of VOCs was based on mass spectra matching with the standard NIST05/Wiley07 libraries (National Institute of Standards and Technology, Gaithersburg, MD, USA), comparing the retention indices (RI) (as Kovats indices) with literature data and from authentic standard compounds when available. The area of individual VOC was determined by the total ion chromatogram (TIC) and semi-quantified via relative comparison with the peak area of the IS (relative peak area, RPA %). Each sample was analyzed in triplicate with a randomized sequence in which blanks were also run.

### 2.9. Statistical Analysis

The PC software “Excel Statistics” version 365 was used for calculations. Results were expressed as the mean ± SD of three experiments. One-way analysis of variance (ANOVA) with a high confidence level (95%, *p* < 0.05) was applied to evaluate and compare the differences between samples. Student’s *t*-tests were applied to examine the mean values of the results. 

## 3. Results and Discussion

Honey has always been considered a sustaining factor in medical treatments. In recent years, some research has ascertained its protective effect as an anti-inflammatory agent and its ability to fight diseases involving the central nervous system, such as Alzheimer’s and Parkinson’s diseases [35,37]. Under such an aspect, honey’s versatile benefits could be due to both its antioxidant activity and its ability to raise the levels of brain-derived neurotrophic factors and the concentration of acetylcholine and, concurrently, to decrease the AChE activity [35]. They could also be dure to the presence of vitamin C and other metabolites, such as polyphenols, which, as well as being antioxidant and anti-inflammatory agents, could be a key font of cholinesterase fighters. Honey contains other molecules, such as volatile compounds, which also perform antioxidant and anti-inflammatory activities; thus, benefits for people’s physiological and psychological health are known [36,37,38,39,40]. Plant VOCs, in particular, can relieve anxiety, cognitive disorders, and depression, maintain memory in patients with Alzheimer’s disease or other memory disorders, and have shown neuroprotective effects [41]. This study assessed the polyphenol and VOCs amount and composition and the vitamin C content in five Italian leguminous honeys (alfalfa, astragalus, carob, indigo, and sainfoin). Furthermore, the anti-inflammatory and antioxidant activities and their inhibitory effects on three enzymes involved in neurodegenerative diseases (AChE, BChE, and tyrosinase) were evaluated.

### 3.1. Total Polyphenols and Polyphenol Profile

The total polyphenol content in the five legume honeys is shown in Table 1.

Sainfoin honey exhibited a higher total polyphenol content than that found, for example, in thyme, turnip, and sunflower honey; alfalfa honey had a higher total polyphenol content than raspberry honey; however, the other three types of honey exhibited a lower quantity of total polyphenols [42]. The total polyphenol values of alfalfa honey are quite similar to those of orange blossom honey [43]. However, the total polyphenol content present in our samples was lower than that observed by Otmani et al. [44], who found a quantity of total polyphenols of no less than 64 mg GAE/100 g of product in different types of Fabaceae honey from the Algerian territory. However, total polyphenols and other biochemical characteristics of honey are related to the territory [44], the type of plant selected by the bees [45], and the period of pollen collection by bees [46]. 

**Figure 1:**
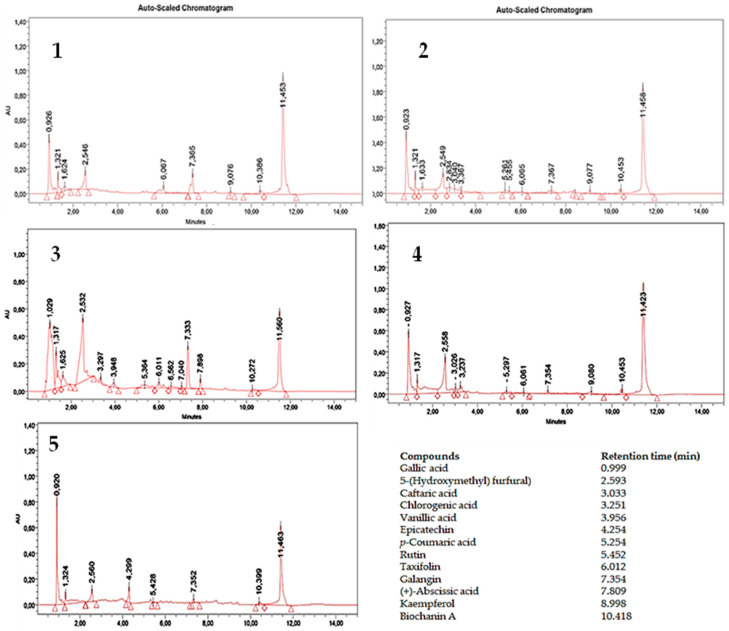
UPLC profile of polyphenols of the five legume honeys: (**1**) astragalus honey; (**2**) indigo honey; (**3**) sainfoin honey; (**4**) alfalfa honey; (**5**) carob honey. In the figure, the retention times of the known polyphenols are also reported.

Figure 1 and Table 2 show the polyphenols’ profile of the five legume honeys, determined through UPLC analysis.

Some metabolites are common to all the samples: gallic acid, 5-hydroxymethyl-furfural (HMF), and galangin; in decidedly smaller quantities, biochanin A as found. All honeys contained high amounts of gallic acid, ranging from 119.64 (indigo honey) to 383.42 μg g^−1^ (carob honey). Gallic acid is one of the phenolic acids frequently found in honeys of different geographic origins [47]. It is the dominant acid in some kinds of honey in Peru, Australia, and New Zealand [48,49]. The amount of gallic acid was different respect that found by Khalil et al. [2], who detected 0.43 mg g^−1^ of gallic acid in tualang honey—one of the most abundant kinds of honey, known for its health properties—and then different types of honey of Bangladesh, which showed an amount of gallic acid not superior to 0.6 mg g^−1^ [50]. The amount of gallic acid found in the five leguminous honeys was also different if compared with that detected in other types of Italian honey, such as acacia, ailanthus, orange, savory, thymus, and chestnut [51], and in the fifty-three Hungarian acacia honey samples analyzed by Matkovits et al. [52]. Our data agree, although partially, with Kara and Kolayli [53], who observed that gallic acid was present in astragalus honey from the Erzincan region of Turkey and rutin was absent; on the contrary, the amount of gallic acid observed by us was lower (206.73 μg g^−1^ of product vs. 42.20 mg 100 g^−1^ of product). Da Silva et al. observed much lower values of gallic acid (18.2–92.7 μg g^−1^) compared to what we observed in legume honey, containing a quantity of gallic acid ranging between 119.64 μg g^−1^ (indigo honey) and 383.42 μg g^−1^ (carob honey) [54]. The presence of gallic acid also suggested the potential role of these honeys as supporting agents with anticancer activity [55]. Therefore, gallic acid has documented action against inflammation and oxidation stress [56], which hare important pathways in carcinogenesis. Galangin was present in all five leguminous honeys, ranging between 5.47 μg g^−1^ (carob honey) and 29.66 μg g^−1^ (astragalus honey). Such values generally higher with those found in the acacia honey (0.04 mg 100 g^−1^ of product) by Oroian and Sorina [57]. Galangin possesses an antioxidative effect on endothelial tissues; thus, it can affect lipid peroxidation and can prevent heart disease. Therefore, it helps preserve other protective antioxidants, such as vitamin E, vitamin C, and other flavonoids, and can prevent lipid peroxidation [58,59]. With other honey polyphenols, galangin acts against cancer through caspases 3, 6, 7, 8, and 9, Bax/Bcl2, Bid, cell migration and invasion, signal transducer and activator of transcription 3 (STAT3), IL-6, S, G0/G1 and G2/M phase, Ki- 67, PCNA, NF-ƘB1, 2, RelA (p65), p-IƘB-α, IƘB-β p-Erk, p-c-Jun N-terminal kinase (JNK), p-p38, ferritin expression, p21, p27, p53, and HO-1 [60]. The level of HMF found in the five honeys ranged between 7.6 μg g^−1^ (carob honey) and 36.52 μg g^−1^ (sainfoin honey). This level falls within the values found for other types of legume honey of Italian origins, such as acacia honey, which, in the studies conducted by Apriceno et al., ranged between 7 and 103 mg kg^−1^ [61]. Generally, HMF arises through sugar degradation through the Maillard reaction (a non-enzymatic browning reaction) during food processing or prolonged honey storage [62]. HMF concentration is a parameter that influences honey freshness; it is typically absent (or is present in only minimal amounts) in fresh honey. In contrast, its concentration tends to rise during processing and because of honey aging. The amount of this metabolite found in the five honeys was lower than those indicated by the *Codex Alimentarius* Standard Commission, which established the maximum limit for HMF in honey at 40 mg kg^−1^ (and the maximum limit for HMF in the honey originating from tropical regions at 80 mg/kg); this limit would ensure that the product would not undergo extensive heating during processing and is safe for consumption [63,64]. 

The taxifolin concentrations found in the five legume honeys were similar to those observed by Jaeger et al. in Fabaceae [65] and by Da Silva et al. [54] in many kinds of honey, including some derived from legume flowers. Taxifolin, found in all types of honey, is an important molecule for the gut microbiome. Treatment with taxifolin has proven helpful in alleviating some types of ulcerative colitis, acting on the gut microbiome and inducing it to produce a greater quantity of butyric acid [66]. It can, among other things, improve any alterations of the intestinal epithelial barrier [67]. All leguminous honeys also contained little biochanin A (BCA), ranging between 2.01 μg g^−1^ (astragalus honey) and 9.81 μg g^−1^ of product (alfalfa honey). BCA is an isoflavone mainly observed in red clover. Other types of honey contain BCA, such as clover [68] and *Castanopsis* honey, which belong to the Fagaceae family [69], and some types of Latvian honey [70]. This molecule shows poor solubility, but it is known to act as a metabolic modulatory agent on glucose and lipids and is estrogen-like; it also has an anti-inflammatory effect and can exhibit cancer prevention activity, as well as neuroprotective and drug interaction effects [71]. Recent studies have demonstrated that the biochanin-A’ anticancer and antiangiogenic activity structure–function relationship is similar to that of the well-known anticancer compound resveratrol and its metabolic product, piceatannol, in breast cancer cells [72]. 

Other secondary metabolites were present in one or more types of honey; kaempferol, for instance, was detected in alfalfa, indigo, and astragalus honeys to a much lesser extent. The polyphenolic portion of alfalfa, indigo, and sainfoin honey contained chlorogenic acid. Alfalfa and indigo honey contained caftaric acid. On the other hand, some metabolites were present in only one kind of honey. Thus, vanillic acid was one of the secondary metabolites in sainfoin honey. Therefore, from a compositional point of view, indigo honey (with eleven metabolites) and alfalfa honey (with nine recognized elements) proved to be the richest honeys. Sainfoin honey and carob honey exhibited seven metabolites identified through UPLC analysis. Astragalus honey was the “poorest”. The content of some of these metabolites, such as vanillic acid (13.4 μg g^−1^ of product), agreed with other types of honey from the Mediterranean area. For example, Splioti et al. [73] analyzed twelve types of honey of Greek origin and found vanillic acid contents between 135 mg kg^−1^ and 701 mg kg^−1^ of product. In our investigation, however, we did not find protocatechuic acid, which, in the types of honey analyzed by Spilioti et al., came to be present at μg g^−1^ concentrations, nor did we find the presence of hydroxybenzoic acid. Evidently, as for the plant world and honey, the polyphenolic component could be influenced by the type of flower that supplies pollen to the bees and the different metabolic pathways that affect the polyphenols themselves. Ramananuskene et al. [74] found a negligible vanillic and gallic acid level. In contrast, they found 3.67 μg mL^−1^ of *p*-coumaric acid in honey obtained from another legume, the acacia. Can et al. reported a quantity of gallic acid of 0.29 μg g^−1^ in astragalus honey. In the same samples, however, they observed an amount of vanillic acid of 1.95 μg g^−1^ of the sample, which we have observed, albeit in absolutely higher quantities, in sainfoin honey [75]. 

### 3.2. Vitamin C Content

The ascorbic acid content of the five legume honeys was highly variable (Table 1), oscillating between 1.61 mg/100 g^−1^ (astragalus honey) and 27.79 mg 100 g^−1^ of product (alfalfa honey). Although astragalus honey had a very low content of ascorbic acid compared to the other types of honey, this was nevertheless higher than that observed in acacia honey (0.77 mg 100 g^−1^ and 0.99 mg 100 g^−1^), as reported by Dobrinas et al. [76]. The ascorbic acid content of the honey samples we analyzed was higher—except astragalus honey—even than that observed by Alshammari et al. [77], and it was in line, if not superior to, that observed by Bouuddine et al. [78]. 

### 3.3. Antioxidant Activity

The antioxidant activity of the five legume honeys, determined using the DPPH test and the ABTS assay, showed a certain variability (Table 1). Sainfoin honey exhibited the most effective radical scavenging capacity (IC_50_ = 6.08 mg mL^−1^). The data obtained by the ABTS assay (10.614 mM TEAC g^−1^ of product) corroborated such a result. Carob honey was second in antioxidant efficacy (IC_50_ = 19.26 mg mL^−1^ and 4.325 mmol TEAC g^−1^ of product). Astragalus, alfalfa, and indigo honeys were the weakest; however, the IC_50_ values were lower than 70 mg mL^−1^. The antioxidant activity of sainfoin honey was in line with that of other kinds of honey, which are well known for their health properties, such as tualang honey [79] and gelam [80]. Our data indicate that, as regards sainfoin honey, it exhibited a better antioxidant activity even than Tamarisk honey [81]. Furthermore, in the case of alfalfa honey, the product analyzed by us, with an IC_50_ value = 58.98 mg mL^−1^, exhibited a better radical scavenging activity than that reported by Abdallah et al. [81], who found an IC_50_ value of no less than 80 mg mL^−1^ for this type of honey. Our results, however, showed that astragalus honey had a weaker radical scavenging activity compared to that found by Gul and Pehlivan [82]; this could, therefore, be attributable not so much to the harvest year—or, in any case, not only to the year—but to the area of origin of the flowers used by the bees in the production of honey.

### 3.4. Anti-Inflammatory Activity

In all organisms, proteins are an essential class of biological macromolecules, whose structure can, however, be modified and altered by the presence of certain compounds, such as free radicals, which, in some cases, can change their structure, therefore inhibiting their normal functions [83]. The anti-inflammatory activity of the five legume honeys was determined by calculating the mg of product necessary to prevent the denaturation of a model protein, BSA, by 50% (IC_50_) and by comparing its behaviour with a conventional anti-inflammatory molecule, diclofenac. 

The results are shown in Table 1. The IC_50_ values varied between 23.7 mg (astragalus honey) and 61.59 mg (alfalfa honey). In any case, two other types of honey analyzed, sainfoin and carob honeys, gave IC50 values relatively close to that of astragalus honey (30.19 mg and 35.27 mg, respectively). Indigo honey, on the other hand, with a value of IC50 of 55.60 mg, exhibited behaviour closer to alfalfa honey. Such results, although deriving from in vitro tests, seem highly comforting. A teaspoon can contain approximately 2 g, if not more, of honey, a quantity of product that would therefore be able to exert the anti-inflammatory action much more effectively than the synthetic molecule used in our experiments. The results are in line with, if not superior to, with those exhibited by some *Citrus* kinds of honey (for example, lime honey and lemon honey) [35]. However, they were less effective than those obtained by Zaidi et al. [84], who observed various monofloral and multi-floral kinds of honey exhibiting an anti-inflammatory efficacy very similar to that of ibuprofen used as a control anti-inflammatory drug. However, in this case, we must remember the different anti-inflammatory efficacies exhibited by diclofenac and ibuprofen, which act at different concentrations (diclofenac at 50 mg kg ^−1^ and ibuprofen at 10 mg kg^−1^ when used, for instance, in evolutionary age for the treatment of headache) (https://www.pharmamedix.com last access 7 February 2024).

### 3.5. Inhibitory Activity of the Legume Honeys against Cholinesterases and Tyrosinase

#### 3.5.1. Activity of the Legume Honeys against Cholinesterases

Some neurodegenerative diseases, such as Alzheimer’s disease, lead to the decay of of acetylcholine and choline concentrations. Concurrently, the AChE enzyme concentration increases markedly, and problems with the potential breakdown of cholinergic synapses and cellular nerve dysfunction can happen [12]. Such a situation, which decreases cholinergic or neurotransmitter activity in the central nervous system, can cause a successive injury to brain memory and cognitive function. Thus, inhibiting the enzyme cholinesterase can help prevent and treat AD. Most studies on brain function have focused on γ-aminobutyric acid (GABA), considered the primary inhibitory neurotransmitter of the central nervous system [85]. The investigation of the activity of AChE and BChE is of clear relevance since, in the late stages of Alzheimer’s disease, levels of AChE decline by up to 85%, and BChE becomes the predominant cholinesterase in the brain [12,13,14,86].

Our study investigated the five leguminous honey’s in vitro perspective inhibitory effect on ACHe and BCHe. Our results, shown in Table 1, indicate that the action of the indigo honey was more effective against BChE, with percentages of inhibition up to 27.4%. Astragalus honey caused an inhibition of 13.4%; on the other hand, sainfoin honey inhibited BChE at 9.24%. Alfalfa honey and carob honey were ineffective. The action of the honeys against the acetylcholinesterase, AChE, was weaker. Sainfoin exhibited the same inhibitory power (9.1%). Indigo honey, the most effective in inhibiting BChE, was ineffective against AChE; astragalus and sainfoin honeys exhibited similar AChE inhibition, ranging between 5.04% (sainfoin) and 6.2% (astragalus). The cholinesterase-inhibitory activity exhibited by the honey has been reported in different in vitro studies. Baranowska-Wójcik et al. analyzed 47 kinds of Polish honeys, finding an AChE-inhibitory activity reaching 39.06%. The results also confirmed that the divergence observed among the types of honey could be due to their different botanical origins [37]. Loizzo et al. ascertained that fig honey showed AChE and BChE IC_50_ values of 46.0 µg mL^−1^ and 46.8 µg mL^−1^, respectively [87]. In vivo studies showed that the administration of 20% of acacia honey effected a decrease in the AChE level from 533.67 to 302.45 mM/min/g of tissue/mg protein × 10^−2^ in the brain of rats [88].

#### 3.5.2. Inhibitory Activity of Legume Honey against Tyrosinase

The neurodegenerative process also takes place in the presence of an excess of tyrosinase. Some studies report that its overexpression can determine the onset of PD19 when a particular threshold value of neuromelanin is exceeded. Consequently, the decrease in the level of intracellular neuromelanin, which can also be determined by an inhibitory action against the tyrosinase enzyme, can contribute to the prevention of neurodegenerative phenomena, limiting—or, at least, delaying—the onset and severity of another significant neurodegenerative disease, Parkinson’s disease. We evaluated whether the five honeys from legumes could inhibit tyrosinase. To assess any inhibitory tyrosinase activity, we carried out two tests. In the first case, tyrosine was used as a substrate (expressing the results in terms of IC_50_); in the second test, L DOPA was used, and the results were expressed in terms of the percentage of inhibition. As a positive control, 0.05 mM of kojic acid was utilized. Kojic acid is one of the most well-known tyrosinase inhibitors and is also utilized in medicine to treat some skin disorders [89]. In the test performed with tyrosine, all types of honey acted as tyrosinase inhibitors (Table 1). Interestingly, in the DOPA test, the IC_50_ values did not exceed 16.23 mg (sainfoin honey); astragalus honey exhibited the best anti-tyrosinase activity, with an IC_50_ value of 10.26 mg. In the test using tyrosine as substrate, the inhibition percentages ranged between 13% (indigo honey) and 74% (sainfoin honey). Considering that kojic acid, with 0.05 mM, gave rise to an inhibitory activity of about 46%, it is evident that the types of honey studied, except indigo, could exhibit a similar inhibitory strength to kojic acid (astragalus honey) or more than 1.5 times stronger (sainfoin honey). The IC_50_ values found in our work indicate that, in many ways, the tyrosinase-inhibitory action was also superior to that exhibited by *Citrus* honey [35] and other types of Thai honey, such as that obtained from coffee [90].

### 3.6. VOCs Profile of Honey

Ninety-seven volatile compounds were identified and semi-quantified via HS SPME GC-MS analysis in the five monofloral Italian legume honey belonging to distinct chemical classes: terpenoids and norisoprenoids (26 compounds), alcohols (14), aldehydes (12), acids (11), esters (9), phenols (6), ketones (5), furans (5), pyranones (3), benzene derivates (2), lactones (2), and sulfur compounds (2). The recognized 97 volatile metabolites, the abbreviation code, the experimental and literature Kovats index, and the identification methods are listed in Table S1. T. SPME GC–MS semi-quantitative data were subjected to one-way ANOVA to investigate the effect of the five honey types on the identified VOCs. Table 3 summarizes the results, showing the 97 volatiles and their semi-quantitative data (RPA%), evaluated as the percent ratio relative to the peak area of 1-octanol, used as IS, obtained via SPME GC–MS analysis. The total number of compounds detected for each legume honey varied among samples, which were 64 for astragalus honey, 60 for sainfoin and indigo honey, 53 for alfalfa, and 45 for carob honey.

Most of the compounds identified were previously recognized in different kinds of honey of diverse geographical origins and obtained via various extraction and detection approaches. Twenty-five VOCS were common to all five legume honeys: nonanal (Ald4); benzaldehyde (Ald6); benzene acetaldehyde (Ald8); 2-ethyl-1-hexanol (A7); 2-furanmethanol (A8); benzyl alcohol (A11); phenyl ethyl alcohol (A12); dl-limonene (T3); *p*-cymene (T6); p-cymenene (T7); cis linalool oxide (T8); trans linalool oxide (T10); linalool (T11); hotrienol (T14); α-terpineol (T16); thymol (T23); 5-isoprenyl-2-methyl-2-vinyl tetrahydrofuran (Herboxide: F3); 2-acetylfuran (F5); acetic acid (Ac1); butanoic acid (Ac3); octanoic acid (Ac6); nonanoic acid (Ac7); benzoic acid (Ac9); dimethyl disulphide (S1); 2,4-di-tert-butylphenol (Ph3) (Table 3). The volatile profile of carob, alfalfa, and sainfoin honeys is mainly composed of terpenes and norisoprenoides, ranging from 38% of the total peak area of alfalfa honey to 90% of the total peak area of carob honey (sainfoin honey: 71.31%) (Table 3). Among terpenes, hotrienol (T14) shows the highest content, accounting for 52.98 and 38.36% of all the terpenoid peak areas in sainfoin and carob honey, respectively (Table 3). The primary terpene compound present in the alfalfa honey sample is cis-linalool oxide (T8), with a content of 14.05% of the total terpene area. T8 is also strongly present in carob honey, with a content equal to 30.67% of the total terpenoid peak area (Table 3). Consistent with the literature data, among the most common honey terpenes, linalool derivatives, such as cis- and trans-linalool oxides, linalool, and hotrienol, are the most abundant. Hotrienol, which gives a sweet flower aroma reminiscent of the plant of origin, was already identified in other studies among the terpenic compounds present in significantly high concentrations in different types of honey (lavender, rhododendron, chestnut, carob, heather, citrus, thyme, eucalyptus, and wild flora). T14 and T8 are compounds derived from honey’s thermal degradation, although studies report their occurrence without prolonged storage or heat treatment [91,92].

The percentage of terpenoids in the honey of astragalus and indigo remains high (around 26%). However, for the astragalus honey, the class of compounds most present is aldehydes (around 31%), while for indigo honey, acids (around 27%) are most present (Table 3).

The VOCs responsible for the versatile biological activity of honey mainly belong to the class of terpenes and isoprenoids, which are well represented in the samples we studied. In particular, the antiradical activity of honey has been linked to norisoprenoids, which are efficient free radical scavengers and improve the vertebrate immune system as they possess potential antioxidant capacity [27,93]. In addition to showing antimicrobial properties and preserving beneficial microorganisms such as lactic acid bacteria, various terpenes also exert antioxidant and antitumor activities [41,91,93]. In particular, there are data for α-pinene (T1) and linalool (T11) suggesting their effects on diseases, including stroke, ischemia, neuropathic pain, cognitive impairment (relevant to Alzheimer’s disease and aging), anxiety, and depression [41]. Medicinal value in the treatment of anxiety syndromes has also been reported for dl-limonene (T3) and α-terpineol (T16) [94]. For thymol (T23), a potent anti-inflammatory agent, a specific cognitive enhancement activity has also been suggested in a rat model with dementia [95]. Furthermore, p-cymene (T6) acted as a potential anti-inflammatory agent in cytokine production by blocking NF-kB and MAPK signalling pathways [95].

Acids in all samples constitute 26.85% and 17.16% of the total peak area in the indigo and astragalus samples, respectively. For the other three honeys analyzed, the percentage of acids ranged from 7.66 of the total peak area of the alfalfa sample to 2.82% of carob honey (Table 3). Acetic acid (Ac1), which is present in all five honeys, was found at significant levels in indigo and astragalus honey (16.93% and 5.63% of the total acid area, respectively) (Table 3). This compound, which gives spicy flavors and aromas, has previously been reported as one of the major volatile components of astragalus honey, and in several other kinds of honey, such as buckwheat, chestnut, acacia, pine, eucalyptus, lime tree, fir tree, lavender, honeydew, orange, rape, rhododendron, thyme, rosemary, and sunflower [92].

Formic acid (Ac2), present at significant levels in indigo and astragalus honeys, is antibacterial. In contrast, butanoic acid (Ac3) can inhibit colon cancer and inflammation-mediated ulcerative colitis [95]. Finally, benzoic acid (Ac9) has shown antifungal activity, inhibiting the growth of moulds, yeasts, and some bacteria [95].

Aldehyde compounds represented 31.42% and 27.49% of the total peak area in astragalus and alfalfa honeys, respectively. In the volatile profile of sainfoin and indigo honeys, the percentage of aldehydes varies from 11.22% to 17.18%, respectively, while these compounds are present in very low amounts in carob honey (0.62%) (Table 3). Among aldehydes, the main constituent for astragalus (15.38% of the total aldehyde area) and indigo (7.01%) samples is Ald12, 5-hydroxymethyl-2-furfural (5HMF), a heterocyclic organic compound that occurs naturally in sugar-containing fresh foods, including milk and honey. The main constituents of alfalfa honey were two aldehydes considered ubiquitous in many other kinds of honey, Ald6 (benzaldehyde) and Ald5 (2-furfural), each representing about 8% of all aldehyde’s peak areas (Table 3). Ald12, produced during food pasteurization and cooking due to dehydration of certain sugars, is also formed during extended food storage under acidic conditions that favour its generation [96]. Therefore, the high percentage of Ald12 in astragalus and indigo honey can be correlated to the significant presence of acids that can catalyze fructose and glucose to form 5-HMF in honey. Alcohols represented 20.59 and 13.26% of the total peak area in alfalfa and indigo honey, respectively. In the volatile profile of astragalus and sainfoin honeys, the percentage of alcohols was around 6%, while for carob honey, it was very low (0.92%). Significant levels of phenyl ethyl alcohol (A12), existent in all kinds of honey, were found in indigo and sainfoin samples (6.17% and 4.59% of the total alcohol peak area, respectively) (Table 3). With a floral odor, this aromatic compound has previously been reported as contributing to the aroma of a wide variety of European honeys [97]. In the volatile profile of indigo honey, 2-furan methanol (A8) was also present (about 5% of the total alcohol peak area), which is a furan-derived compound considered, like Ald5, to be a potential indicator of honey spoilage due to poor storage conditions or thermal processes [95]. For alfalfa samples, among the alcohols, the principal constituent (4.48% of the total alcohol peak area) is A11 (benzyl alcohol), a benzene derivative reported—as well as Ald6 (benzaldehyde) and phenyl ethyl alcohol (A12)—in many honeys with different floral origins [98]. Honey volatile compounds, such as 2-furfural (Ald5), benzaldehyde (Ald6), benzene acetaldehyde (Ald8), 2-furan methanol (A8), or phenyl ethyl alcohol (A12), have shown antimicrobial activity [92,95].

Ester compounds, utterly absent in alfalfa and indigo honey, are predominant in sainfoin honey from the point of view of number (8), even if they represent only 2% of the total peak area. Ethyl benzoate (E6) shows the highest content, accounting for 0.64% of the total ester peak area. On the other hand, methyl salicylate (E7), an ester absent in the honey of sainfoin was found only in carob and astragalus honey (0.06% and 0.75% of the total ester area, respectively) (Table 3). The latter ester is effective in treating muscle pain, back pain, and other rheumatic conditions [95]. Phenol compounds constituted about 1% of the total peak area in indigo honey, approximately 0.8% in astragalus and alfalfa honey, and about 0.4% in carob and sainfoin honey (Table 3). Amongst phenols, the main constituent of the five honeys (between 0.1% and 0.6% of the total phenol area) was Ph3 (2, 4-di-tert-butylphenol), a bioactive compound with antibacterial, anti-inflammatory and antioxidant properties reported in different groups of plants and also in honey [99]. On the other hand, methoxy eugenol (Ph6), a phenolic compound naturally found in spices and herbs, was detected only in carob samples (0.24% of the total phenol area), being absent in the other samples (Table 3).

Ketone compounds represented 4.62%, 2.19%, and 1.2% of the total peak area in carob, astragalus, and indigo honey, respectively. On the other hand, these compounds were present in low and minimal quantities in alfalfa and sainfoin honey (0.87% and 0.03% of the total peak area, respectively). In the volatile profile of carob honey, significant levels (4.55% of the total ketones peak area) of a ketone previously reported at high concentrations in the volatile composition of Greek thyme honey, the 3-hydroxy-4-phenyl-2-butanone (K5), were found. This volatile compound is absent in the other four honey samples [100] (Table 3).

In the volatile profile, furans represented approximately 2% of the total peak area in astragalus honey, about 1% in sainfoin, alfalfa, and indigo samples, and only 0.3% in carob honey (Table 3).

Finally, pyranones, significant volatile compounds in honey due to their potential health benefits, were absent in carob honey and detected in very small percentages (between 0.18% and 0.66% of the total volatile) in sainfoin and alfalfa honey. They constitute about 12% of the total peak area in indigo and astragalus samples. Among pyranones, the main constituent for astragalus (10.54% of the total pyranones area) and indigo (10.94%) samples was Pyr-2-(2,3-dihydro-3,5-dihydroxy-6-methyl-4H-pyran-4-one, DDMP), reported in several food products, including dried plums and honey, which has been associated with both antioxidant activity and the ability to stimulate the activities of the autonomic nerves of rats [101,102] (Table 3).

### 3.7. Correlation Analysis

The correlation analysis did not highlight an apparent influence of the total polyphenols on the cholinesterase-inhibitory activities exhibited by the five legume honeys, except ACHe, where a moderate influence exerted by the total content of these metabolites was noted (ρ = 0.46). This partially agrees with Szwajgier [40], who noted a correlation between total polyphenol content and cholinesterase-inhibitory activity (ρ = 0.33 for TPC/AChE; ρ = 0.48 for TPC/BChE). A naturally more incisive influence was noted in the antioxidant activity. In this case, the effect of total polyphenols was evident, so much so that we recorded a correlation value of ρ = 0.93 in the TEAC test compared to ρ = −0.91 in the DPPH test, where, as we know, the antioxidant efficacy is measured in terms of IC_50_. The correlation coefficients indicated a more significant correlation between AChE-inhibitory action and antioxidant activity (DPPH and TEAC), so much so that the correlation coefficient found between AChE-inhibitory activity and TEAC was 0.67, and the correlation coefficient between AChE-inhibitory activity and DPPH was ρ = −0.14. The correlation between antioxidant activity and BChE-inhibitory activity (IC_50_) was much more pronounced; here, the BChE and TEAC correlation coefficients were equal to 0.95, while the BChE and DPPH correlation coefficients were equal to −0.74. As regards the inhibitory action on tyrosinase, we observed a good correlation with the presence of total polyphenols, which seemed to have influenced the inhibitory activity of tyrosinase on L tyrosine more (ρ = 0.861), compared to that exerted by tyrosinase on L DOPA (ρ = −0.47). The good influence exerted by total polyphenols on the ability to inhibit tyrosinase confirms—indeed, reinforces—what has already been described by Boutoub et al. [103], in whose work, studying Euphorbia honey, a correlation value between TPC and tyrosinase-inhibitory activity of ρ = 0.56 was noted. The anti-inflammatory activity instead seemed to be influenced more by the presence of vitamin C (ρ = 0.65); on the contrary, total polyphenols appeared to influence it negatively (ρ = −0.45). Compared to the quantity of individual polyphenols, at least those most present in the various types of honey, the correlation analysis highlights a strong influence of gallic acid on the tyrosinase-inhibitory activity of honey on L-tyrosine (ρ = 0.84). This also agrees with Fadzil et al. [10], who noted a good neuroprotective effect of gallic acid and quercetin in honey. Instead, the metabolite HMF seemed to affect, above all, the AChE-inhibitory action of honey (ρ = 0.84). When present in the honey, rutin and taxifolin seemed to influence the honey’s inhibitory action, calculated as a percentage of BChE (ρ = 0.80 and 0.75, respectively). Therefore, flavonoids, such as rutin and taxifolin, can communicate with specific signalling pathways and adjust their activities accordingly, prompting valuable neuroprotective impacts [20,21,22,23,24,25,26,27,28,29,30,31,32,33,34,35,36,37,38,39,40,41,42,43,44,45,46,47,48,49,50,51,52,53,54,55,56,57,58,59,60,61,62,63,64,65,66,67,68,69,70,71,72,73,74,75,76,77,78,79,80,81,82,83,84,85,86,87,88,89,90,91,92,93,94,95,96,97,98,99,100,101,102,103,104]. The antioxidant activity seemed to be influenced, above all, by the presence of HMF; it influenced the antioxidant activity with ρ = of 0.83 (compared to TEAC) and −0.33 (compared to DPPH). The anti-inflammatory activity was more influenced by the presence of gallic acid (ρ = −0.57).

A comparison of our in vitro results with the literature could confirm the role of specific molecules, or classes of molecules, in the biological properties (antioxidant, anti-inflammatory, neuroprotective) exhibited by particular foods [105,106].

Here, we wanted to consider not only polyphenols, a family of molecules widely studied in honey, but also the volatile components, often overlooked in studies of honey biochemistry, but which can give valuable information both on the specificity of a product and on their role in combating diseases that lead to a general decline in the physiological conditions of the CNS. The evaluation of the influence exerted by the various classes of VOCs on biological activities, allowed us to underline the following: (1) that esters seem to have influenced, above all, the inhibitory action of honey on AChE (ρ = 0.83), the anti-inflammatory activity (ρ = −0.62) and, albeit more weakly, the antioxidant activity, calculated in IC_50_ with the DPPH method (ρ = −0.59). Therefore, a previous work reported the influence of some types of honey and their compounds, including esters, on the inflammatory events occurring in rats and mice [107]; (2) that acids, lactones, pyranones, and phenols influenced the inhibitory action of honey on BChE (ρ = 0.94, 0.89, 0.83, and 0.72, respectively). Nordin et al. [108] reported that typical honey, containing approximately 8.57 meq/kg lactone, could protect against neurodegenerative disease, including its inhibitory activity on BChE. Terpenoids and norisoprenoids exerted a good influence on the inhibitory action of honey on tyrosine in the test carried out with tyrosine as a substrate (ρ = 0.83), as well as on the antioxidant activity evaluated with the DPPH test and expressed in terms of IC_50_ (ρ = −0.86). The analysis identified a strong correlation between the VOCs only and the inhibitory activity of the legume honeys against BChE. We ascertained a strong influence of the alcohols A8 and A12, the acetic acid (ρ = 0.899), and the phenolic compound (Ph3) (ρ = 0.82) on the inhibitory activity on BChE (based on the results of the test performed using L-tyrosine). On the other hand, the furan derivate F3 (herboxide) seemed to affect the inhibitory activity on BChE when evaluated through the DOPA (ρ = −0.91). A8 was also found in the essential oil of *Artemisia macrocephala*, with potential inhibitory activity against AChE and BChE [109]. Based on our knowledge, this is the first time that a certain influence of some VOCs on the BChE-inhibitory action exhibited by the five legume honeys—and, perhaps, honey in general—has been hypothesized.

## 4. Conclusions

Plant-derived bioactive compounds have received more interest in recent years than synthetic bioactive compounds in the treatment of many diseases, including neurodegeneration. Thus, through the selection of suitable plant-derived bioactive compounds, plant formulations, or even specific foods, including honey, the standard therapies could be modified or optimized. Bioactive compounds can exert incalculable potential, as proven by their capacity to influence the expression and activity of numerous proteins implicated in oxidative stress and neuroinflammation [110]. As indicated by the results, the legume honeys studied herein showed interesting biochemical characteristics and intriguing in vitro biological properties. As already seen in the case of other monofloral honeys, such as *Citrus* honey, their potential inhibiting action against the three most important enzymes involved in neurodegenerative diseases makes them appealing candidates for implementation in recommended diets or in pharmacological treatment to fight some NDDs, especially in the older adults. A correlation analysis could provide more information about the influence of a specific type of vegetal or vegetal-derived food (in our case, honey) on specific diseases. The chemical composition could enable new scenarios in treating different diseases; there is a close relationship of dependence between the qualitative–quantitative profile of bioactive molecules present and the role (and the influence) that specific plant or plat-derivative products can exert in combating certain pathologies. Thus, by combining the molecules or classes of molecules with the biological properties of such foods, it is possible to formulate a “precision diet” specifically designed to fight certain diseases, including those of the CNS. In the case of the five Italian legume honeys, although supported only by in vitro studies, it is possible to hypothesize that when gallic acid and HMF are concurrently present, honey could represent a potentially important natural source, coupled, eventually, with conventional drugs, in the treatment of neurodegenerative diseases such as Alzheimer and Parkinson due to its inhibitory action against AChE and tyrosinase. Such an action could be reinforced by other molecules, such as esters. The presence of rutin and taxifolin, with a concurrently high amount of some volatile molecules such as some alcohols lactones, pyranones, phenols, or the furan derivate F3, could contribute to fighting AD through the inhibition of BChE. In any case, in addition to classes of molecules or single molecules, it is also worth underlining the fact that a strong correlation between the antioxidant activity of honey and its neuroprotective effects has been detected, confirming the fact that most pathologies derive from oxidation and inflammation events that affect cells and the organism, leading to degenerative mechanisms. Future steps will be to determine the action of these honeys against other enzymes involved in dysmetabolic pathologies, such as α-glucosidase and the α-amylase. The results will be helpful in defining the *in vivo* activity of these types of honey on human health.

## Figures and Tables

**Table 1:** Biochemical characterization of the five legume honeys. Results are the average of three independent experiments (±SD). For details, see the Materials and Methods section. nd: not detected.

	Indigo	Astragalus	Alfalfa	Carrob	Sainfoin
Total polyphenols (μg GAE g^−1^)	110 ± 11	145 ± 8	177 ± 9	291 ± 12	408 ± 19
DPPH (IC_50_, mg mL^−1^)	47 ± 13	59 ± 9	59 ± 14	19 ± 2	6 ± 1
TEAC (mM TE g^−1^)	2.0 ± 0.9	2.4 ± 0.4	4 ± 2	4.3 ± 0.5	10.6 ± 0.1
Vit C (mg/100 g)	17 ± 1	1.6 ± 0.3	27.8 ± 0.9	15.1 ± 0.3	23.7 ± 0.9
Anti-inflammatory activity (IC_50_, mg)	56 ± 8	24 ± 4	62 ± 2	35 ± 7	30 ± 5
AChE-inhibitory activity (%)	nd	6.2 ± 0.3	5.0 ± 0.2	nd	9.1 ± 0.4
AChE-inhibitory activity (IC_50_, mg)	nd	141 ± 13	180 ± 14	nd	112 ± 10
BchE-inhibitory activity (%)	27 ± 3	13 ± 2	nd	0.5 ± 0.1	9 1
BchE-inhibitory activity(IC_50_, mg)	nd	nd	nd	nd	130 ± 13
Tyrosinase-inhibitory activity (DOPA, IC_50_, mg)	13 ± 1	10 ± 1	16 ± 2	11 ± 1	15 ± 2
Tyrosinase-inhibitory activity (%, using L-tyrosine)	13 ± 1	41 ± 3	55 ± 5	71 ± 2	74 ± 2

**Table 2:** Profile of polyphenols recognized in the five types of the legume honey through UPLC analysis. The data are reported as μg g^−1^ of product.

	Astragalus	Carob	Alfalfa	Indigo	Sainfoin
	μg g^−1^	μg g^−1^	μg g^−1^	μg g^−1^	μg g^−1^
**Gallic acid**	206.73	383.42	174.64	119.64	291.18
**5-(Hydroxymethyl)** **Furfural**	14.29	7.6	25.01	13.27	36.52
**Caftaric acid**			11.39	24.06	
**Chlorogenic acid**			25.18	16.82	13.4
**Vanillic acid**					14.43
**Epicatechin**		18.42			
***p*** **-Coumaric acid**			7.98	6.36	
**Taxifolin**	20.52		14.36	22.23	13.89
**Rutin**		2.33		13.77	
**Galangin**	29.66	5.47	6.23	9.63	12.33
**(+)-Abscissic acid**			3.01		
**Kaempferol**	1.80		11.17	6.08	
**Biochanin A**	2.01	4.14	9.81	6.66	4.42

**Table 3:** VOCs identified in the five legume honeys.

Metabolites	Code	Carob	Alfalfa	Sainfoin	Astragalus	Indigo	*p*
**Esters**							
Ethyl butanoate	**E1**	nd	nd	3.69	nd	nd	***
Ethyl 3-methylbutanoate	**E2**	nd	nd	1.03	nd	nd	***
Ethyl hexanoate	**E3**	nd	nd	4.34	nd	nd	***
Ethyl 3-hexenoate	**E4**	nd	nd	6.76	nd	nd	***
Ethyl octanoate	**E5**	nd	nd	2.65	nd	nd	***
Ethyl benzoate	**E6**	nd	nd	12.75	nd	nd	***
Methyl salicylate	**E7**	4.64	nd	nd	10.15	nd	***
Ethyl phenylacetate	**E8**	nd	nd	7.32	nd	nd	***
Methyl 3,5-dimethoxybenzoate	**E9**	nd	nd	1.95	0.09	nd	***
**Aldehydes**							
2-Methyl- 2 butenal	**Ald1**	nd	18.93	6.63	5.15	4.60	***
3-Methyl- 2 butenal	**Ald2**	nd	12.61	nd	nd	nd	***
Octanal	**Ald3**	10.7	nd	5.67	7.11	3.06	**
Nonanal	**Ald4**	8.65	14.24	18.80	18.09	45.47	***
2-Furfural	**Ald5**	nd	79.83	25.21	27.32	48.16	**
Benzaldehyde	**Ald6**	25.97	81.29	58.47	30.90	75.86	*
5-Methyl-2-furfural	**Ald7**	nd	5.35	0.59	25.11	20.81	*
Benzene acetaldehyde	**Ald8**	4.30	36.61	85.88	83.16	7.65	*
5-Formylfurfural	**Ald9**	nd	7.77	16.77	20.00	10.0	*
4-Methoxy benzaldehyde	**Ald10**	nd	2.04	nd	nd	nd	***
3-Phenylpropenal	**Ald11**	nd	1.85	0.00	0.00	8.89	***
5-Hydroxymethyl-2-furfural (5HMF)	**Ald12**	nd	4.75	4.79	207.96	154.77	**
**Alcohols**							
3-Methyl-1-butanol	**A1**	23.38	nd	6.96	nd	nd	***
3-Methyl- 3-buten-1-ol	**A2**	nd	21.24	5.86	3.64	2.48	***
2-Methyl-2-buten-1-ol	**A3**	nd	7.68	1.17	nd	3.06	***
*trans*-3-Hexen-1-ol	**A4**	nd	1.58	nd	nd	nd	***
*cis*-3-Hexene-1-ol	**A5**	nd	35.76	nd	nd	nd	***
1-Octen-3-ol	**A6**	nd	12.34	nd	nd	nd	***
2-Ethyl-1-hexanol	**A7**	9.36	26.27	10.00	13.42	24.70	*
2-Furanmethanol	**A8**	0.88	12.14	7.96	50.19	109.67	***
1 Nonanol	**A9**	7.29	nd	nd	nd	nd	***
5-Methyl-2-furanmethanol	**A10**	nd	nd	nd	0.48	4.46	***
Benzyl alcohol	**A11**	17.86	43.26	7.79	6.14	12.12	***
Phenyl ethyl alcohol	**A12**	14.42	37.44	91.08	14.66	136.23	**
3-Phenyl-2-propen-1-ol	**A13**	nd	0.96	nd	nd	nd	***
4-Methoxy phenethyl alcohol	**A14**	nd	nd	nd	3.61	nd	***
**Terpenoids & Norisoprenoids**							
α-Pinene	**T1**	nd	2.04	nd	0.51	nd	**
α-Terpinene	**T2**	nd	nd	nd	1.01	0.84	*
dl-Limonene	**T3**	2.00	2.68	0.98	1.38	1.75	*
*p*-Mentha-1,5,8-triene	**T4**	nd	nd	nd	nd	1.17	**
γ-Terpinene	**T5**	nd	nd	nd	0.68	nd	*
*p*-Cymene	**T6**	1.61	2.87	0.71	2.15	3.31	*
*p*-Cymenene	**T7**	3.89	17.12	2.94	11.10	52.32	***
*cis*-Linalool oxide	**T8**	2437.05	135.6	144.41	71.34	186.29	****
Nerol oxide	**T9**	57.43	nd	12.77	nd	nd	***
*trans*-Linalool oxide	**T10**	387.17	101.98	94.68	45.52	74.14	**
Linalool	**T11**	123.81	10.42	31.19	38.41	14.56	***
Lilac aldehyde	**T12**	11.55	nd	1.22	13.61	nd	**
Edulan	**T13**	nd	nd	0.28	25.40	nd	**
Hotrienol	**T14**	3047.60	46.40	1052.0	105.38	169.45	****
Ketoisophorone	**T15**	nd	nd	nd	7.65	nd	**
α-Terpineol	**T16**	10.29	8.98	5.96	9.46	5.74	*
Epoxylinalol	**T17**	90.54	nd	3.66	nd	nd	***
β-Damascenone	**T18**	690.52	nd	8.46	5.08	6.56	****
*p*-Cymen-8-ol	**T19**	nd	31.44	nd	2.30	29.47	**
2,6-Dimethyl-3,7-octadien-2,6-diol	**T20**	229.97	nd	56.11	4.09	7.40	***
Safranal	**T21**	nd	nd	nd	5.05	0.40	**
3,7-Dimethyl-1,7-octadien-3,6-diol	**T22**	4.10	nd	nd	nd	nd	*
Thymol	**T23**	0.30	5.70	0.50	0.17	9.64	*
Carvacrol	**T24**	nd	1.80	nd	nd	6.66	**
2,6-Dimethyl-2,7-octadiene-1,6-diol	**T25**	60.08	nd	nd	nd	nd	***
*trans*-Isoeugenol	**T26**	4.84	nd	nd	1.74	nd	*
**Furans**							
3 Methyl furan	**F1**	9.56	nd	nd	nd	nd	***
2-Pentylfuran	**F2**	nd	nd	6.00	nd	nd	***
5-Isoprenyl-2-methyl-2-vinyl tetrahydrofuran (Herboxide)	**F3**	10.33	0.20	4.68	15.85	4.49	**
Anethofuran	**F4**	nd	nd	nd	5.39	3.24	**
2-Acetylfuran	**F5**	7.44	8.64	11.22	7.17	11.06	*
**Acids**							
Acetic acid	**Ac1**	36.59	28.31	25.88	76.17	373.77	***
Formic acid	**Ac2**	nd	nd	nd	31.79	116.80	***
Butanoic acid	**Ac3**	5.70	2.95	21.40	4.83	3.02	***
3-Methylbutanoic acid	**Ac4**	34.17	6.5	17.17	24.50	nd	**
Hexanoic acid	**Ac5**	nd	nd	13.95	17.44	5.68	**
Octanoic acid	**Ac6**	29.70	11.63	16.09	19.80	17.66	***
Nonanoic acid	**Ac7**	111.17	16.07	24.84	23.20	23.54	***
Decanoic acid	**Ac8**	nd	2.82	5.10	11.35	9.11	**
Benzoic acid	**Ac9**	4.05	5.61	13.24	16.18	32.38	***
Dodecanoic acid	**Ac10**	nd	nd	nd	3.04	6.21	***
Phenylacetic acid	**Ac11**	2.59	nd	1.37	3.66	4.74	*
**Sulfur Compounds**							
Dimethyl disulfide	**S1**	2.54	23.97	0.51	0.72	3.92	***
Dimethyl trisulfide	**S2**	nd	1.46	nd	nd	3.89	***
**Ketons**							
3-Hydroxy-2-butanone	**K1**	5.84	0.91	nd	0.79	nd	***
2-Hydroxy-3-methyl-2-cyclopenten-1-one	**K2**	nd	nd	nd	nd	5.82	***
1-(3-Hydroxy-2-furanyl) ethanone	**K3**	nd	7.49	0.35	27.25	20.60	**
4-Hydroxy-3-methylacetophenone	**K4**	nd	nd	0.18	1.50	nd	***
3-hydroxy-4-phenyl-2-butanone	**K5**	361.56	nd	nd	nd	nd	***
**Benzene Derivatives**							
Toluene	**B1**	3.98	1.58	1.30	nd	1.05	***
Benzyl nitrile	**B2**	1.35	nd	nd	5.71	2.88	***
**Lactones**							
2(5H)-Furanone	**L1**	nd	nd	nd	2.57	5.25	**
3-Hydroxy-4,4-dimethyldihydro-2(3H)-furanone	**L2**	nd	2.30	1.82	1.60	27.55	***
**Pyranones**							
Maltol	**Pyr1**	nd	nd	nd	13.60	7.00	**
2,3-Dihydro-3,5-dihydroxy-6-methyl-4h-pyran-4-one (DDMP)	**Pyr2**	nd	6.37	3.5	142.42	241.68	***
5-Hydroxymaltol	**Pyr3**	nd	nd	nd	5.81	10.96	**
**Phenols**							
2,6-Di-tert-butyl-4-methylphenol	**Ph1**	nd	1.88	nd	nd	nd	***
2-Methoxy-4-vinylphenol	**Ph2**	5.47	1.84	1.62	nd	8.74	**
2,4-Di-tert-butylphenol	**Ph3**	4.75	2.55	2.17	4.09	11.51	**
3,4,5-Trimethylphenol	**Ph4**	nd	nd	3.06	6.28	3.89	**
4-Methoxyphenol	**Ph5**	nd	0.90	nd	nd	nd	*
Methoxyeugenol	**Ph6**	18.79	nd	nd	nd	nd	**

Mean values of three samples are calculated as RPA (%). Data are expressed as the mean of three experiments; nd: not detected. For each metabolite, the mean values, followed by different number of asterisks, are significantly different according to one-way ANOVA test (*, **, ***, **** = significant at values < 0.05, 0.01, 0.001, and 0.0001, respectively).

## Data Availability

The data presented in this study are available on request from the corresponding author.

## Supplementary Materials

The following supporting information can be downloaded at: https://www.mdpi.com/article/10.3390/antiox13040482/s1, Table S1: Volatile metabolites detected in the five legume honeys and their identification codes.
