# Supplementary material for: Chemical Characterization and Biological Properties of Leguminous Honey
Source: Antioxidants (Basel). 2024 Apr 18;13(4):482. doi: 10.3390/antiox13040482 (PMC11047671; doi:10.3390/antiox13040482)
Supplement: Supplementary file 1 [file antioxidants-13-00482-s001.zip › antioxidants-2957432-supplementary.pdf]

Table S1. Volatile metabolites detected in the five legume honeys and their identification codes

Metabolite	Code	RIt/RIsp	ID	Metabolite	Code	RIt/RIsp	ID
**Esters**				Epoxylinalol	T17	1753/1752	RI/MS/S
Ethyl butanoate	E1	1028/1028	RI/MS/S	β-Damascenone	T18	1805/1805	RI/MS/S
Ethyl 3-methylbutyrate	E2	1054/1054	RI/MS/S	p-Cymen-8-ol	T19	1838/1839	RI/MS/S
Ethyl hexanoate	E3	1248/1249	RI/MS/S	2,6-Dimethyl-3,7-octadien-2,6-diol	T20	1948/1949	RI/MS
Ethyl 3-hexenoate	E4	1304/1316	RI/MS	Safranal	T21	1648/1977	MS/S
Ethyl octanoate	E5	1434/1434	RI/MS/S	3,7-Dimethyl-1,7-octadien-3,6-diol	T22	2134/2134	RI/MS
Ethyl benzoate	E6	1647/1647	RI/MS/S	Thymol	T23	2183/2182	RI/MS/S
Methyl salicylate	E7	1766/1766	RI/MS/S	Carvacrol	T24	2217/2217	RI/MS/S
Ethyl phenylacetate	E8	1770/1770	RI/MS	2,6-Dimethyl-2,7-octadiene-1,6-diol	T25	2344/2349	RI/MS
Methyl 3,5-dimethoxybenzoate	E9	-	MS/S	*trans*-Isoeugenol	T26	2372/-	MS/S
**Aldehydes**				**Furans**			
2-Methyl- 2 butenal	Ald 1	1100/1099	RI/MS/S	3 Methyl furan	F1	901/901	RI/MS/S
3-Methyl- 2 butenal	Ald 2	1236/1242	RI/MS/S	2-Pentylfuran	F2	1237/1238	RI/MS/S
Octanal	Ald 3	1307/1307	RI/MS/S	5-Isoprenyl-2-methyl-2-vinyl tetrahydrofuran (Herboxide)	F3	1253/1255	RI/MS
Nonanal	Ald 4	1398/1399	RI/MS/S	Anethofuran	F4	1484/1497	RI/MS
2-Furfural	Ald 5	1468/1469	RI/MS/S	2-Acetylfuran	F5	1506/1506	RI/MS/S
Benzaldehyde	Ald 6	1527/1527	RI/MS/S	**Acids**			
5-Methyl-2-furfural	Ald 7	1570/1569	RI/MS/S	Acetic acid	Ac1	1460/1460	RI/MS/S
Benzene acetaldehyde	Ald 8	1628/1628	RI/MS	Formic acid	Ac2	1515/1514	RI/MS/S
5-Formylfurfural	Ald 9	1991/1991	RI/MS	Butanoic acid	Ac3	1610/1610	RI/MS/S
4-Methoxy benzaldehyde	Ald 10	2035/2035	RI/MS/S	3-Methylbutanoic acid	Ac4	1653/1653	RI/MS/S
3-Phenylpropenal	Ald 11	2049/2050	RI/MS/S	Hexanoic acid	Ac5	1834/1835	RI/MS/S
5-Hydroxymethyl-2-furfural (5HMF)	Ald 12	2532/-	MS/S	Octanoic acid	Ac6	2058/2058	RI/MS/S
**Alcohols**				Nonanoic acid	Ac7	2165/2165	RI/MS/S
3-Methyl-1-butanol	A1	1240/1243	RI/MS/S	Decanoic acid	Ac8	2320/2320	RI/MS/S
3-Methyl- 3-buten-1-ol	A2	1277/1283	RI/MS/S	Benzoic acid	Ac9	2448/-	MS/S
2-Methyl-2-buten-1-ol	A3	1333/1345	RI/MS/S	Dodecanoic acid	Ac10	2515/-	MS/S
trans-3-Hexen-1-ol	A4	1394/1397	RI/MS/S	Phenylacetic acid	Ac11	2570/-	MS/S
cis-3-Hexene-1-ol	A5	1400/1400	RI/MS/S	**Sulfur compounds**			
1-Octen-3-ol	A6	1456/1457	RI/MS/S	Dimethyl disulfide	S1	1063/1063	RI/MS/S
2-Ethyl-1-hexanol	A7	1494/1494	RI/MS/S	Dimethyl trisulfide	S2	1376/1376	RI/MS/S
2-Furanmethanol	A8	1646/1647	RI/MS/S	**Ketones**			
1 Nonanol	A9	1649/1649	RI/MS/S	3-Hydroxy-2-butanone	K1	1314/1315	RI/MS/S
5-Methyl-2-furanmethanol	A10	1711/1710	RI/MS	2-Hydroxy-3-methyl-2-cyclopenten-1-one	K2	1818/1818	RI/MS
Benzyl alcohol	A11	1869/1868	RI/MS/S	1-(3-Hydroxy-2-furanyl) ethanone	K3	2001/2000	RI/MS
Phenyl ethyl alcohol	A12	1906/1905	RI/MS/S	4-Hydroxy-3-methylacetophenone	K4	2180/2194	RI/MS/S
3-Phenyl-2-propen-1-ol	A13	2334/2368	RI/MS/S	3-hydroxy-4-phenyl-2-butanone	K5	2260/2310	RI/MS
4-Methoxy phenethyl alcohol	A14	2355/-	MS/S	**Benzene derivatives**			
**Terpenoids & Norisoprenoids**				Toluene	B1	1035/1036	RI/MS/S
α-Pinene	T1	1019/1019	RI/MS/S	Benzyl nitrile	B2	1920/1921	RI/MS/S
α-Terpinene	T2	1174/1174	RI/MS/S	**Lactones**			
dl-Limonene	T3	1205/1205	RI/MS/S	2(5H)-Furanone	L1	1742/1741	RI/MS/S
p-Mentha-1,5,8-triene	T4	1210/1213	RI/MS/S	3-Hydroxy-4,4-dimethyldihydro-2(3H)-furanone	L2	2028/2028	RI/MS/S
γ-Terpinene	T5	1231/1232	RI/MS/S	**Pyranones**			
p-Cymene	T6	1277/1277	RI/MS/S	Maltol	Pyr1	1961/1962	RI/MS/S
p-Cymenene	T7	1438/1438	RI/MS/S	2,3-Dihydro-3,5-dihydroxy-6-methyl-4h-pyran-4-one (DDMP)	Pyr2	2311/2315	RI/MS
cis Linalool oxide	T8	1446/1448	RI/MS/S	5-Hydroxymaltol	Pyr3	2309/2374	RI/MS
Nerol oxide	T9	1466/1467	RI/MS/S	**Phenols**			
trans Linalool oxide	T10	1473/1473	RI/MS/S	2,6-Di-tert-butyl-4-methylphenol	Ph1	1927/2101	RI/MS/S
Linalool	T11	1542/1543	RI/MS/S	2-Methoxy-4-vinylphenol	Ph2	2195/2195	RI/MS/S
Lilac aldehyde	T12	1573/1571	RI/MS/S	2,4-Di-tert-butylphenol	Ph3	2330/-	MS/S
Edulan	T13	1602/1584	RI/MS/S	3,4,5-Trimethylphenol	Ph4	2248/-	MS/S
Hotrienol	T14	1596/1596	RI/MS/S	4-Methoxyphenol	Ph5	-	MS/S
Ketoisophorone	T15	1675/1674	RI/MS/S	Methoxyeugenol	Ph6	2534/-	MS/S
α-Terpineol	T16	1684/1684	RI/MS/S				

RI_t: Relative retention indices on polar column reported in literature by www.pherobase.com; www. flavornet.org; www.ChemSpider.com; webbook.nist.gov; pubchem.ncbi.nlm.nih.gov; **RI_{sp}**: Relative retention indices calculated against n-alkanes (C_8-C_{40}) on HP-Innowax column; **ID:** Identification method as indicated by the following: RI: Kovats retention index on the HP-Innowax column; MS: NIST05 and Wiley07 libraries spectra; S: co-injection with authentic standard compounds, where commercially available, on the HP-Innowax column.
